# Supplementary material for: Effect of the COVID-19 pandemic on drug-resistant tuberculosis treatment outcomes at a national referral hospital in Sierra Leone, 2017 to 2022: A retrospective cohort study
Source: PLOS Glob Public Health. 2026 Mar 4;6(3):e0004472. doi: 10.1371/journal.pgph.0004472 (PMC12959693; doi:10.1371/journal.pgph.0004472)
Supplement: Attachment — Submitted filename: DR TB point to point response to comment.docx [file pgph.0004472.s003.docx]

**Point-by-Point Response to Reviewers**

**Manuscript ID:** PGPH-D-25-00640
**Title:** Effect of the COVID-19 pandemic on drug-resistant tuberculosis treatment outcomes in Sierra Leone, 2017 to 2022: a national retrospective cohort study

**Reviewer #1:**

**Comment 1:** *"One of the main comments is the elaboration of a table explaining the logistic regression variables with their respective adjusted ORs and confidence intervals."*

**Response:** We thank the reviewer for this excellent suggestion. We have now included added **Table 4** in the manuscript that presents the full results of the logistic regression analysis.

**Reviewer #2:**

**Major Comment 1: Study design is mis-classified.**

- **Comment:** "Re-define the work as a retrospective cohort study..."
- **Response:** We agree entirely with the reviewer. Our study design is indeed a **retrospective cohort study**.
- **Actions Taken:**
  - **Abstract:** Changed from "cross-sectional retrospective analysis" to "**retrospective cohort study**".
  - **Methods:** The "Study Design" subsection now begins: "We conducted a **retrospective cohort study**...".
  - **Manuscript:** The title has been updated to "...a national retrospective cohort study" to reflect the national scope of the database.
  - **STROBE Checklist:** Updated to adhere to the STROBE guidelines for cohort studies.

**Major Comment 2: Confounding by treatment regimen and calendar time.**

- **Comment:** *"Include regimen type... Add calendar year (or quarter) to control for secular trends."*
- **Response:** This was a crucial point. Our re-analysis confirms the reviewer's concern about confounding.
- **Actions Taken:**
  1. **Final Adjusted Model:** We added treatment regimen (Short vs. Long) and calendar year (as a continuous variable) to the final multivariable logistic regression model. The results are in **Table 4**.
  2. **Key Finding:** The results can be found in lines 211-233 of the manuscript with tracked changes.
  3. **Effect Modification:** We tested an interaction between study period and treatment regimen. The interaction term was not statistically significant (**p = 0.793), indicating that** the effect of the pandemic period did not differ by regimen type.
  4. **Clarification on Regimens:** We have clarified that the newer BPaL-M regimen was introduced after our study period (Dec 2023). The "short" regimen in our analysis refers to the older standardised shorter MDR-TB regimen introduced in October 2020.
  5. **Manuscript Changes:** We have completely revised the **Abstract, Results, and Discussion** to base all inferences on this final adjusted model and emphasize the role of confounding.

**Major Comment 3: Model diagnostics and robustness.**

- **Comment:** *"Report VIFs... Hosmer-Lemeshow... C-statistic... pattern of missingness..."*
- **Response:** We have now performed and reported these diagnostics (lines 161 to 164).
- **Actions Taken:**
  - **Multicollinearity:** The Variance Inflation Factor (VIF) for all variables was below 2.0, indicating no concerning multicollinearity.
  - **Goodness-of-fit:** The Hosmer-Lemeshow test indicated adequate fit (p = 0.78).
  - **Discrimination:** The area under the ROC curve (C-statistic) was 0.62.
  - **Missing Data:** We excluded 38 patients (5.1%) due to missing data. The pattern was random. A sensitivity analysis using best-case/worst-case scenarios confirmed our primary conclusions were robust. This is now described in the Methods.

**Major Comment 4: Interpretation overstates the evidence.**

- **Comment:** *"Base all inferences on the adjusted model..."*
- **Response:** We sincerely thank the reviewer for this critical correction. We have entirely rewritten the interpretation.
- **Actions Taken:**
  - The **Abstract, Results, and Discussion** now strictly base conclusions on the adjusted model, which shows **no significant effect** of the COVID-19 period.
  - We explicitly acknowledge that the initial unadjusted result was misleading and likely driven by confounding factors.
  - All language claiming a "significant" independent effect of COVID-19 has been removed.

**Major Comment 5: Data availability non-compliant.**

- **Comment:** *"The Figshare link provided is private."*
- **Response:** We apologise for this oversight.
- **Actions Taken:** The dataset has been fully anonymised and uploaded to Figshare under a **public CC-BY license**. The new link is: <https://figshare.com/account/items/30171058/edit> . The **Data Availability Statement** has been updated accordingly.

**Minor Comments:**

- **Typographical errors:** Corrected "resitant" to "resistant" and "peroid" to "period" throughout.
- **Abbreviations:** All abbreviations (e.g., DR-TB, BPaL-M, aOR) are now defined at first use. A dedicated **Abbreviations** section has been added.
- **Clarifications:** We have added requested details on the number of excluded cases (n=38), specified the time periods more clearly, and added mean ages and standard deviations for age groups.
- **Reviewer Comment:** *“Sierra Leone is one of the 30 high TB burden countries …where?”*
- **Response:** Thank you for highlighting this. We have clarified that Sierra Leone is among the 30 high tuberculosis (TB) burden countries in the world, as designated by the World Health Organization. This classification is based on global epidemiological data reflecting TB incidence and mortality rates.
- **Comments***: from the National DR-TB Database covering multiple facilities. Please align title, setting and Methods.”*
- **Author Response:**
  We thank the reviewer for this important comment, which has helped us improve the clarity of our methodology. The reviewer is correct that the National DR-TB Database contains data from multiple facilities across Sierra Leone.
- However, for this study, we extracted a specific subset of this national database: **only the data for patients who were diagnosed and managed at the National TB Referral Hospital (Lakka Government Hospital)**. This hospital is the primary site for DR-TB treatment in the country, and its data is routinely sent to the national database for programmatic monitoring and decision-making.
- To eliminate any ambiguity and fully align the title, setting, and methods as suggested, we have revised the **Study Setting** and **Methods** sections to explicitly state that:
- The study population consists of patients from the National TB Referral Hospital.
- The *source* of the data for these patients is the National DR-TB Database.
- We believe these clarifications ensure that the manuscript now accurately reflects that the study's scope is indeed the national referral hospital, and the national database was the mechanism for accessing this single-center data
- **Reviewer Comment :**
- *“Specify how many cases were excluded?”*
- **Response:** We appreciate this important suggestion. The methodology section has been updated to specify that 38 of the 739 recorded patients were excluded due to ongoing treatment or missing key characteristics. As a result, 701 cases were included in the final outcome analysis. (Lines 151 to 164)
- **Reviewer Comment :**
- *“I suggest specifying the variables pre-COVID-19 and post-COVID-19 in relation to TB treatment.”*
- **Response:** Thank you for this helpful recommendation. We have now clearly defined three distinct time periods—pre-COVID-19, during COVID-19, and post-COVID-19—and categorized treatment outcomes for each as either successful (cured, treatment completed) or unsuccessful (death, loss to follow-up, treatment failure, and not evaluated). This structure enhances the clarity and comparability of our findings.
- **Reviewer Comment:**
- *“Deidentified to Identified”*
- **Response:** Thank you for raising this point. We would like to clarify that all patient-level data used in this study were fully de-identified prior to extraction from the National Drug-resistant TB Database on 24 September 2023. No personally identifiable information was accessed or included at any stage of the analysis. This approach aligns with national ethical standards and data protection protocols. The manuscript has been revised to reflect this more clearly.

We believe these revisions have substantially improved our manuscript, and we thank the reviewers again for their time and expertise.
