# Supplementary material for: Effect of the COVID-19 pandemic on drug-resistant tuberculosis treatment outcomes at a national referral hospital in Sierra Leone, 2017 to 2022: A retrospective cohort study
Source: PLOS Glob Public Health. 2026 Mar 4;6(3):e0004472. doi: 10.1371/journal.pgph.0004472 (PMC12959693; doi:10.1371/journal.pgph.0004472)
Supplement: Attachment — Submitted filename: DR TB response to reviwers 1_1_26.docx [file pgph.0004472.s004.docx]

**Manuscript ID:** PGPH-D-25-00640
**Title:** Effect of the COVID-19 pandemic on drug-resistant tuberculosis treatment outcomes in Sierra Leone, 2017 to 2022: a national retrospective cohort study

Reviewer #1:

Comments: I think it's a good article with the statistical rigor necessary for understand this important topic

Response: Thank you!

Reviewer #2:

Comment 1: Thank you for the opportunity to review the revised version of PGPH-D-25-00640_R1. The manuscript has improved substantially: the study is now correctly framed as a retrospective cohort, the statistical methods have been strengthened with additional adjustment for calendar year and regimen type and with appropriate model diagnostics and sensitivity analyses, and the interpretation of the association between the COVID-19 period and treatment outcomes has been suitably tempered. Overall, the work is methodologically sound, clearly reported and of clear relevance for global TB control.

Response: Thank you!

Comment 2: I have only minor remaining concerns, mainly relating to data sharing and small editorial details: the Figshare link provided in the Data Availability Statement still points to a private “account/edit” URL and therefore does not comply with PLOS’s open-data policy; I encourage the authors to deposit a fully anonymised dataset with an accessible public DOI (including a data dictionary and, if possible, analysis code) and to cite that DOI in the manuscript.

Response: Dear Reviewer, thank you for requesting the data. I sincerely apologize for sharing a link that is private. The delay in re-submitting this work was to enable us to publish the data in an online repository that can be accessed by the reviewers. We attempted to publish the data in Dryad, but the holidays created challenges that prevented us from paying. We have now been able to publish the data in fig share data repository with a publicly available link ([*https://doi.org/10.6084/m9.figshare.30986815*](https://doi.org/10.6084/m9.figshare.30986815)*)*. The data uploaded in this link included the raw data in an excel sheet and the coded data available in the SPSS sheet (Line 327 of the clean version and line 329 in the version with tracked changes).

Comment 3: In addition, I suggest a final consistency check to ensure that the design is uniformly described as a retrospective cohort throughout, that all abbreviations (e.g. DR-TB, BPaL-M, aOR) are defined at first use, and that minor typographical issues and formatting of percentages and confidence intervals are corrected. Once these small points—particularly the public data link—are addressed, I consider the manuscript suitable for publication in PLOS Global Public Health and recommend Minor Revision.

Response: Thank you. We have made minor corrections and editing where they are necessary.
